# Urban particulate matter stimulation of human dendritic cells enhances priming of naive CD8 T lymphocytes

**DOI:** 10.1111/imm.12852

**Published:** 2017-11-28

**Authors:** Paul E. Pfeffer, Tzer R. Ho, Elizabeth H. Mann, Frank J. Kelly, Maria Sehlstedt, Jamshid Pourazar, Rosamund E. Dove, Thomas Sandstrom, Ian S. Mudway, Catherine M. Hawrylowicz

**Affiliations:** ^1^ MRC and Asthma UK Centre for Allergic Mechanisms of Asthma King's College London Guy's Hospital London UK; ^2^ Environmental Research Group MRC‐PHE Centre for Environment and Health King's College London London UK; ^3^ NIHR Health Protection Research Unit in Health Impact of Environmental Hazards Faculty of Life Sciences and Medicine King's College London London UK; ^4^ Division of Medicine Department of Public Health and Clinical Medicine Umeå University Umeå Sweden; ^5^ Present address: William Harvey Research Institute Queen Mary University of London London EC1M 6BQ UK

**Keywords:** CD8^+^ T lymphocyte, dendritic cells, granulocyte–macrophage colony‐stimulating factor, granzyme, lung, particulate matter

## Abstract

Epidemiological studies have consistently shown associations between elevated concentrations of urban particulate matter (UPM) air pollution and exacerbations of asthma and chronic obstructive pulmonary disease, which are both associated with viral respiratory infections. The effects of UPM on dendritic cell (DC) ‐stimulated CD4 T lymphocytes have been investigated previously, but little work has focused on CD8 T‐lymphocyte responses despite their importance in anti‐viral immunity. To address this, we examined the effects of UPM on DC‐stimulated naive CD8 T‐cell responses. Expression of the maturation/activation markers CD83, CCR7, CD40 and MHC class I on human myeloid DCs (mDCs) was characterized by flow cytometry after stimulation with UPM *in vitro* in the presence/absence of granulocyte–macrophage colony‐stimulating factor (GM‐CSF). The capacity of these mDCs to stimulate naive CD8 T‐lymphocyte responses in allogeneic co‐culture was then assessed by measuring T‐cell cytokine secretion using cytometric bead array, and proliferation and frequency of interferon‐*γ* (IFN‐*γ*)‐producing T lymphocytes by flow cytometry. Treatment of mDCs with UPM increased expression of CD83 and CCR7, but not MHC class I. In allogeneic co‐cultures, UPM treatment of mDCs enhanced CD8 T‐cell proliferation and the frequency of IFN‐*γ* ^+^ cells. The secretion of tumour necrosis factor‐*α*, interleukin‐13, Granzyme A and Granzyme B were also increased. GM‐CSF alone, and in concert with UPM, enhanced many of these T‐cell functions. The PM‐induced increase in Granzyme A was confirmed in a human experimental diesel exposure study. These data demonstrate that UPM treatment of mDCs enhances priming of naive CD8 T lymphocytes and increases production of pro‐inflammatory cytokines. Such UPM‐induced stimulation of CD8 cells may potentiate T‐lymphocyte cytotoxic responses upon concurrent airway infection, increasing bystander damage to the airways.

**Abbreviations**
anova: analysis of variance
BAL: bronchoalveolar lavage
BW: bronchial wash
CFSE: 5‐(and ‐6)‐carboxyfluorescein diacetate succinimidyl ester
COPD: chronic obstructive pulmonary disease
DC: dendritic cell
GM‐CSF: granulocyte–macrophage colony‐stimulating factor
IFN‐*γ*: interferon‐*γ*
IL‐2: interleukin‐2
mDC: myeloid dendritic cell
PM: particulate matter
Tc: CD8^+^ T lymphocyte
TNF‐*α*: tumour necrosis factor‐*α*
UPM: urban particulate matter

## Introduction

Exposures to elevated concentrations of ambient particulate matter (PM) have been shown to be associated with increased asthma exacerbations^1^, ^2^, ^3^, ^4^ and viral upper respiratory tract infections^5^, ^6^, ^7^ – these may be linked as most asthma exacerbations are triggered by upper viral respiratory tract infections.^8^, ^9^ Similarly, increased pollution exposure has been associated with exacerbations of chronic obstructive pulmonary disease (COPD),^10^, ^11^ a disease characterized by enhanced susceptibility to respiratory viruses^12^ and infection‐driven airway inflammation.^13^

Dendritic cells (DCs) play a major role in orchestrating immune responses in the lungs^14^, ^15^ and extensive research has studied the effects of urban particulate matter (UPM) on DC‐stimulated CD4 T‐lymphocyte responses.^16^, ^17^, ^18^, ^19^ For example, we have previously shown that UPM treatment of granulocyte–macrophage colony‐stimulating factor (GM‐CSF) ‐stimulated CD1c^+^ myeloid dendritic cells (mDCs) leads to increased proliferation of naive CD4 T lymphocytes in an allogeneic mixed lymphocyte reaction.^16^ However, decreased priming of interferon‐*γ* (IFN‐*γ*) ‐producing lymphocytes was also observed and impaired priming of T helper type 1 responses could compromise anti‐viral immune responses.

CD8 T lymphocytes are of prime importance in anti‐viral adaptive immunity,^12^, ^20^, ^21^, ^22^ yet the effect of UPM on DC‐stimulated CD8 responses has not been similarly studied. CD8 T lymphocytes employ several mechanisms to kill infected or malignant cells: secretion of cytokines such as IFN‐*γ* and tumour necrosis factor‐*α* (TNF‐*α*) that have downstream anti‐viral effects, and the production and release of cytotoxic granules containing perforin and granzymes. However, CD8 T lymphocytes can also secrete a wider panel of cytokines mirroring the well‐described CD4 T‐cell subsets.

Dendritic cells comprise only a small minority of leucocytes but play a critical role in coordinating adaptive immune responses, in part by activating naive T cells and regulating differentiation. The mDCs lie just under the airway epithelium on which ambient PM deposits^23^ and Nizzoli *et al*.^24^ have shown that CD1c^+^ mDCs can potently prime and activate CD8 T lymphocytes.

The lung is a GM‐CSF‐rich environment. GM‐CSF is important for survival of DCs in non‐lymphoid tissue,^25^ and the action of GM‐CSF on pulmonary DCs is influential in lymphocyte responses in the lung.^25^, ^26^ Treatment of epithelial cells with diesel exhaust particles, a major component of UPM, is known to stimulate production of GM‐CSF that in turn promotes DC maturation and activation.^18^ It is therefore pertinent to consider the actions of UPM on CD8 priming in the context of the GM‐CSF‐rich pulmonary environment.

In this study we have investigated the effect of UPM on CD1c^+^ mDC‐stimulated naive CD8 T lymphocytes in the presence and absence of GM‐CSF. Initially we examined the effects of UPM treatment on the expression of mDC maturation/activation markers (CD83 and CD40), the chemokine receptor CCR7 and of MHC class I, which presents antigenic peptides to CD8 T lymphocytes. Ligation of CD40 on DCs has previously been shown to be important for DC stimulation of lymphocytes^27^ and the chemokine receptor CCR7 directs migration of activated mDCs to lymph nodes where they then prime naive lymphocytes. We then ascertained the effect of UPM treatment on DC‐primed naive CD8 T‐lymphocyte responses, to establish whether PM treatment might impair or promote the priming of CD8^+^ cytotoxic T cell type 1 (Tc1) responses.

## Materials and methods

### Cell culture

Peripheral blood was obtained from healthy volunteers after informed consent (NRES Ethics 09/H0804/77). Peripheral blood mononuclear cells were isolated by density centrifugation over Lymphoprep (Axis‐Shield, Alere Technologies AS, Oslo, Norway). CD1c^+^ mDCs were further isolated using a MACS CD1c^+^ DC isolation kit (Miltenyi Biotec, Bergisch Gladbach, Germany) in accordance with the manufacturer's instructions. Purity was assessed as > 98% CD11c^+^ HLA‐DR^+^ by flow cytometry. Cells were cultured in cell culture medium (RPMI‐1640; Gibco, Thermo Fisher Scientific, Waltham, MA, USA) supplemented with 10% fetal bovine serum, 2 mm l‐glutamine and 0·1% 50 mg/ml gentamicin. Then, 50 ng/ml GM‐CSF (R&D Systems, Minneapolis, Minnesota) and/or 5 μg/ml UPM (standard reference material 1648a; National Institute of Standards and Technology (NIST), Gaithersburg, MD) or a vehicle control (5% methanol/ultrapure water) were added to stimulate the cultures. NIST 1648a is an urban PM sample previously collected in the USA and with mean particle diameter 5·85 μm.^28^

Naive CD8 T lymphocytes were isolated from human peripheral blood mononuclear cells through the use of a MACS Naive CD8 Cell isolation kit (Miltenyi Biotec) in accordance with the manufacturer's instructions. Naive CD8 T‐lymphocyte purity was assessed by flow cytometry as (mean average) 91·6% CD8^+^ CD45RA^+^ CCR7^+^ CD56^–^ CD57^–^. To assess subsequent proliferation, naive CD8 T lymphocytes were immediately labelled with 5‐(and ‐6)‐carboxyfluorescein diacetate succinimidyl ester (CFSE; Invitrogen, Thermo Fisher Scientific, Waltham, MA, USA) after isolation in accordance with the manufacturer's instructions. Proliferation was determined by loss in fluorescence intensity in co‐culture. In some experiments anti‐human HLA‐A,B,C antibody (10 μg/ml, BioLegend, San Diego, CA) or mouse IgG2a isotype control (10 μg/ml, BioLegend) were added to cultures.

### MDC flow cytometric analysis

The mDCs were cultured for 20 hr at a cell density of 1 × 10^5^/ml in 96‐well U‐bottom plates with relevant stimuli and were then harvested using ice‐cold 2 mm EDTA/1% fetal bovine serum in PBS (Gibco) before staining with fluorochrome‐labelled antibodies as follows: CD83‐allophycocyanin (BD Bioscience, San Jose, CA), CD40‐FITC (BioLegend), CCR7‐phycoerythrin (BioLegend), HLA‐ABC‐Peridinin chlorophyll protein (BioLegend); or appropriate isotypes. Flow cytometry was performed on Attune Acoustic (Life Technologies) or FACSCalibur (BD Bioscience) flow cytometers.

### mRNA analysis

After 20 hr of cell culture, mDCs were lysed with Qiazol reagent (Qiagen, Hilden, Germany) and homogenized with QIAshredder columns before storage at −80° pending extraction of mRNA using an miRNeasy Mini Kit (Qiagen) according to an adapted manufacturer's protocol with an off‐column DNA digest with TurboDNase (Ambion, Thermo Fisher Scientific, Waltham, MA, USA) and RNeasy MinElute Cleanup Kit (Qiagen). Messenger RNA was quantified using a NanoDrop ND‐1000 Spectrophotometer (ThermoScientific, Thermo Fisher Scientific, Waltham, MA, USA) and converted to cDNA using RevertAid Reverse Transcriptase and complementary reagents (Fermentas, ThermoScientific). Relative quantification of target genes relative to the *18S* housekeeping gene was conducted in triplicates by real‐time quantitative PCR using Taqman Universal PCR MasterMix (Applied Biosystems, Thermo Fisher Scientific, Waltham, MA, USA) and an Applied Biosystems Viia 7 real‐time thermal cycler. Results were analysed using viia 7 software (Applied Biosystems). Taqman primers were purchased from Applied Biosystems.

### Allogeneic co‐cultures

After 20 hr of culture, naive CFSE‐labelled CD8 T lymphocytes from a different donor were then added at a 1 : 5 ratio of DCs to naive CD8 T cells to produce an allogeneic mixed lymphocyte reaction. On day 5 of co‐culture, supernatant was removed and stored at −20**°** for subsequent analysis of cytokine protein concentrations, and an aliquot of T cells was removed for flow cytometric analysis of cell proliferation. The DC : T‐cell co‐cultures were then expanded in fresh media containing 5 U/ml recombinant human interleukin‐2 (IL‐2) (Eurocetus, Harefield, UK) before performing intracellular cytokine staining 2 days later. Cells were stimulated for 2 hr with 5 ng/ml PMA and 500 ng/ml Ionomycin (Sigma‐Aldrich, St Louis, MO) and then 2 μm Monensin was added for a further 2 hr to block cytokine secretion. Cells were incubated with 2 μm of 7‐aminoactinomycin D (7‐AAD; Sigma) to assess their viability before being fixed and permeabilized using BD PermFix (BD Bioscience). The cells were then stained with allophycocyanin‐labelled anti‐IFN‐*γ* (BioLegend) for 40 min at room temperature before wash in Cytofix/Cytoperm buffer and then were analysed on an Attune Acoustic Focusing Cytometer (Applied Biosystems). In some experiments cells were additionally stained with phyoerythrin‐labelled anti‐Granzyme A (BioLegend) and Alexa Fluor 647‐labelled anti‐Granzyme B (BioLegend) but without CFSE labelling. Flow cytometry data were analysed using flowjo (FlowJo LLC; version 10, San Diego, CA).

### Cytometric bead array

The concentration of cytokines in cell culture supernatants was assessed by Cytometric Bead Array multiple cytokine assay (BD Biosciences).

### Human exposure studies

Sixteen healthy non‐smoking volunteers, free from respiratory infection or pre‐existing allergic disease were recruited and exposed on two separate occasions, once to filtered air and once to diesel engine exhaust, with exposures separated by at least 3 weeks to limit carry‐over effects. Each exposure lasted for 1 hr, during which the subjects alternated between 15 min of rest and exercise (20 l/min/m^2^ body surface). Diesel exhaust was generated by an idling Volvo diesel engine Volvo (TD45, 4·5 L, 4 Cylinders, 1991, 680 rpm). This exposure duplicated an earlier protocol used to investigate the pro‐inflammatory nature of diesel exhaust.^29^ The protocol was approved by the local Ethical Review Board at Umeå University, and performed in accordance with the Declaration of Helsinki with written informed consent of all participating volunteers. Further information is given in the Supplementary material (Appendix S1).

Bronchoscopy was performed 6 hr after the diesel and filtered air exposures using a flexible video bronchoscope (Olympus BF IT160, Japan) with proximal and lower airway samples obtained by bronchial wash (BW, 2 × 20 ml) and bronchoalveolar lavage (bronchoalveolar (BAL), 3 × 60 ml) respectively, using sterile saline. Granzyme A was determined in cell‐free BW and BAL fluid samples using a commercial ELISA kit (BioVendor, Brno, Czech Republic). Messenger RNA was generated from BAL leucocytes using the Qiagen RNeasy Mini Kit and the Superscript III First‐Strand Synthesis System for quantitative PCR kit from Invitrogen Technologies (Paisley, UK), following the manufacturer's instructions. Glyceraldehyde 3‐phosphate dehydrogenase (GAPDH) was selected as a reference gene for this data set. BAL cell cytospins were fixed in 2% paraformaldehyde and after washing, were used for immunocytochemistry analysis with a Granzyme A monoclonal antibody (R&D Systems, Minneapolis, Minnesota). Data were expressed as the % positive cells in each cytospin. Further information is shown in the Supplementary material (Appendix S1).

### Statistical analysis

Results were analysed in graphpad prism 6·0 by two‐way repeated‐measures analysis of variance (anova) (unless otherwise stated) with presence/absence of UPM stimulation as the first factor and presence/absence of GM‐CSF as the second factor, with multiple comparisons post‐tests corrected by the Bonferroni method. Secreted cytokine data were normalized by logarithmic transformation before anova analysis. Data are presented as mean ± SEM or as box‐and‐whisker plots with median, interquartile range and absolute range.

## Results

### UPM stimulates enhanced expression of multiple DC activation markers but not expression of MHC class 1

**Figure 1:**
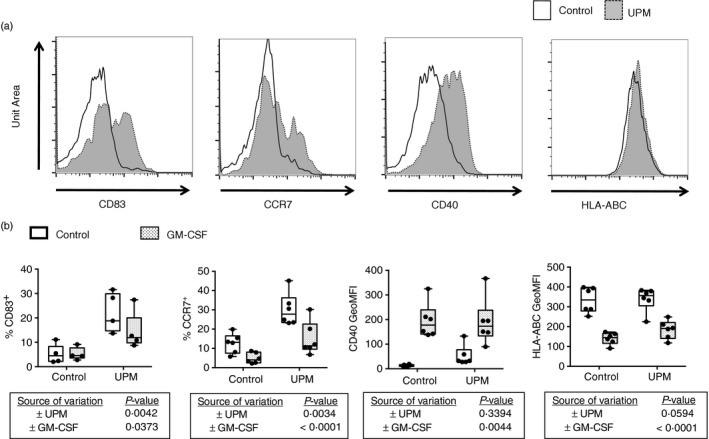
Urban particulate matter (UPM) treatment of CD1c^+^ myeloid dendritic cells (mDCs) enhances expression of CD83 and CCR7. (a) Representative frequency‐histogram flow‐cytometry plots showing effect of 5 μg/ml UPM on CD1c^+^ mDC expression of CD83, CCR7, CD40 and HLA‐ABC after 20 hr of culture. (b) Summary data for effect of UPM with/without 50 ng/ml granulocyte–macrophage colony‐stimulating factor (GM‐CSF) on mDC cultures. Two‐way analyses of variance, *n* = 5 or *n* = 6.

Human CD1c^+^ mDCs were isolated from peripheral blood and cultured for 20 hr with 5 μg/ml UPM, a dose we have previously shown to significantly enhance mDC expression of CD80,^16^ with and without 50 ng/ml GM‐CSF, and expression of other mDC activation markers measured by flow cytometry (Fig. 1a,b). UPM significantly enhanced mDC expression of CD83 (*P* = 0·0042) and CCR7 (*P* = 0·0034). UPM stimulation enhanced CD40 expression compared with unstimulated cultures (*P* = 0·013), but only in the absence of GM‐CSF. In contrast, GM‐CSF significantly decreased mDC expression of CCR7 (*P* < 0·0001) but significantly enhanced CD40 expression (*P* = 0·0044). Expression of MHC class 1 (MHC‐I), as measured by a fluorochrome‐tagged antibody to HLA‐ABC, was not significantly affected by UPM but was suppressed by GM‐CSF (*P* < 0·0001).

Given the novel finding that UPM increased CCR7, expression of a panel of chemokine receptors was analysed by quantitative real‐time PCR – this confirmed the strong enhancement of CCR7 expression upon UPM stimulation of DCs. Notably CCR7 was the only one of the chemokine receptors studied (CCR1, CCR2, CCR5, CCR6, CCR7) to show increased expression with UPM stimulation (see Supplementary material, Fig. S1).

### UPM‐stimulated mDCs enhance proliferation of naive CD8 T lymphocytes in an allogeneic mixed lymphocyte reaction

**Figure 2:**
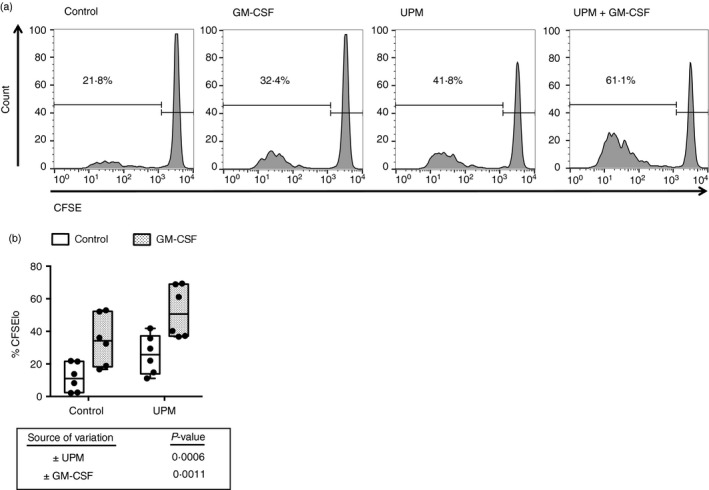
Urban particulate matter (UPM) and granulocyte–macrophage colony‐stimulating factor (GM‐CSF) treatments of myeloid dendritic cells (mDCs) both enhance their ability to stimulate proliferation of naive CD8 T lymphocytes added to cultures. (a) Representative flow‐cytometry plots and (b) summary data showing effect of 5 μg/ml UPM with/without 50 ng/ml GM‐CSF treatment of CD1c^+^ mDCs on their ability to stimulate proliferation at day 5 of naive CD8 T lymphocytes in allogeneic mixed‐lymphocyte reactions, as measured by dilution of CFSE. Two‐way analyses of variance, *n* = 6.

To examine the effect of UPM treatment on DC stimulation of naive CD8 T lymphocytes, CFSE‐labelled naive CD8 T lymphocytes were co‐cultured for 5 days with allogeneic mDCs that had been pre‐cultured in the presence/absence of UPM with/without GM‐CSF for 20 hr. Both GM‐CSF and UPM treatment of mDCs enhanced their stimulatory capacity with resulting increased CD8 T‐cell proliferation (Fig. 2a,b). Maximal CD8 T‐cell proliferation was seen with combined UPM and GM‐CSF treatment of DCs.

### UPM‐stimulated mDCs increase pro‐inflammatory cytokine production by alloreactive CD8 T lymphocytes

**Figure 3:**
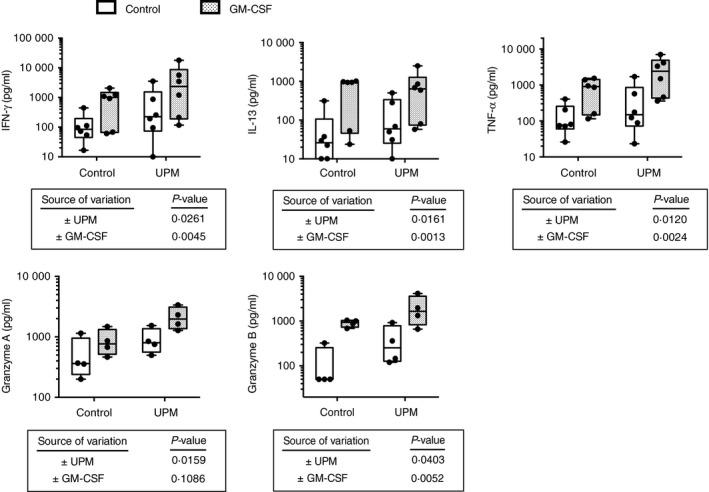
Urban particulate matter (UPM) and granulocyte–macrophage colony‐stimulating factor (GM‐CSF) treatments of myeloid dendritic cells (mDCs) increase the production of pro‐inflammatory cytokines by naive CD8 T lymphocytes in mixed‐lymphocyte reactions. (a) Effect of 5 μg/ml UPM with/without 50 ng/ml GM‐CSF treatment of CD1c^+^ mDCs on the production of pro‐inflammatory cytokines at day 5 in co‐cultures with naive CD8 T lymphocytes in allogeneic mixed‐lymphocyte reactions. Interferon*‐γ* (IFN‐*γ*), *n* = 6; tumour necrosis factor‐*α* (TNF‐*α*), *n* = 6; interleukin‐13 (IL‐13), *n* = 6; Granzyme A, *n* = 4; Granzyme B, *n* = 4. Two‐way analyses of variance after normalization by logarithmic transformation.

The levels of secreted cytokines in DC–CD8 T‐cell co‐culture supernatants harvested at 5 days was measured by Cytometric Bead Array. UPM stimulation of DCs significantly increased production of IFN‐*γ*, TNF‐*α*, IL‐13, Granzyme A and Granzyme B in co‐culture (Fig. 3), whereas GM‐CSF‐pretreated DCs significantly increased production of all these mediators except Granzyme A (*P* = 0·11). The highest levels of the pro‐inflammatory cytokines were seen with the combination of GM‐CSF and UPM. Next, we examined whether UPM stimulation affects the polarity (Tc1 versus Tc2) of alloreactive CD8 T lymphocytes in co‐culture – on analysing the ratio of IFN‐*γ* to IL‐13 production by two‐way anova analysis neither UPM nor GM‐CSF significantly affected the IFN‐*γ*/IL‐13 ratio (data not shown).

**Figure 4:**
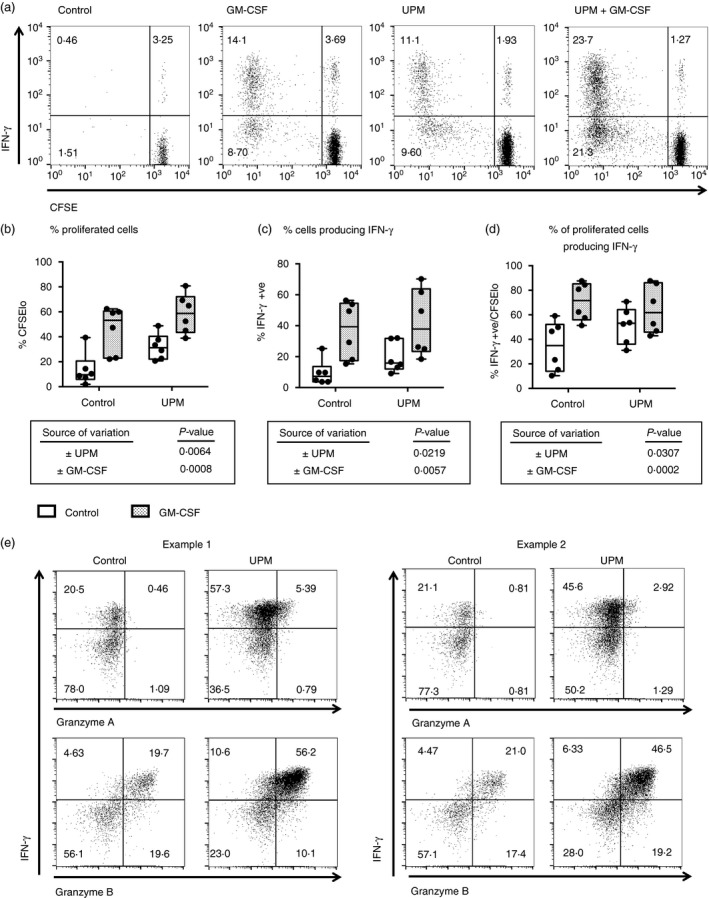
Urban particulate matter (UPM) and granulocyte–macrophage colony‐stimulating factor (GM‐CSF) treatments of myeloid dendritic cells (mDCs) both enhance priming of interferon‐*γ* (IFN‐*γ*) ‐producing CD8 T lymphocytes. (a) Representative flow‐cytometry plots and (b–d) summary data showing effect of 5 μg/ml UPM with/without 50 ng/ml GM‐CSF treatment of CD1c^+^ mDCs on their ability to stimulate proliferation and prime IFN‐*γ* production by CD8 T lymphocytes in allogeneic mixed‐lymphocyte reactions, as measured by dilution of CFSE and intracellular cytokine staining, after 5 days co‐culture and 2 days expansion with interleukin‐2. Two‐way analyses of variance, *n* = 6. (e) Flow‐cytometry plots for effect of UPM on CD8 T‐cell intracellular cytokine staining for Granzyme A and Granzyme B after co‐culture and expansion as above

To further examine the capacity of UPM‐treated DCs to prime Tc1 responses, CFSE‐labelled naive CD8 T lymphocytes at 5 days were then expanded for 2 further days with low dose IL‐2 and priming to produce IFN‐*γ* assessed by intracellular cytokine staining and flow cytometry (Fig. 4). In line with the 5‐day culture results, both UPM and GM‐CSF significantly enhanced proliferation of alloreactive CD8 T lymphocytes (*P* = 0·0064 and *P* = 0·0008; Fig. 4b). The proportions of both total CD8 T lymphocytes (Fig. 4c) and of divided CD8 T lymphocytes (Fig. 4d) that were primed to secrete IFN‐*γ* were significantly increased by UPM stimulation (*P* = 0·022 and *P* = 0·031, respectively) and significantly increased by GM‐CSF (*P* = 0·0057 and *P* = 0·0002). In cultures with GM‐CSF‐treated DCs the proportion of divided CD8 T lymphocytes primed to secrete IFN‐*γ* was not further increased by UPM co‐activation; however, neither did UPM suppress the priming of IFN‐*γ*‐secreting CD8 T lymphocytes. In cultures with DCs that had not been exposed to GM‐CSF, UPM increased the proportion of divided CD8 T lymphocytes primed to secrete IFN‐*γ*.

To better phenotype the UPM‐stimulated naive CD8 T‐lymphocyte response, further experiments were conducted with an enlarged panel of intracellular cytokine staining (Fig. 4e). The majority of IFN‐*γ*‐expressing T cells were Granzyme B co‐expressing, and consistent with the secreted cytokine data UPM stimulation increased the proportion of lymphocytes expressing Granzyme B. Furthermore upon UPM stimulation a subset of Granzyme A expressing CD8 T cells emerged.

**Figure 5:**
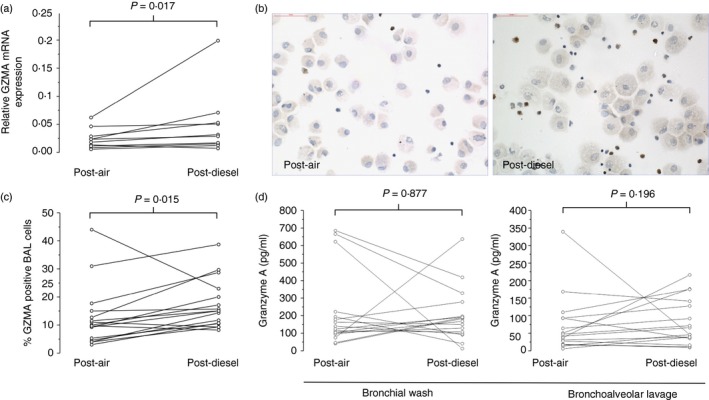
Granzyme A mRNA and protein expression in healthy human volunteers 6 hr after controlled air and diesel exposures. (a) *GRZMA* mRNA expression relative to *GAPDH* in bronchoalveolar lavage (BAL) leucocytes 6 hr post‐air and diesel exposure. (b) Representative cytospin stainings from samples 6 hr post‐ exposure. (c) Granzyme A staining cells as a percentage of the leucocytes within cytospins. (d) Granzyme A concentrations in the cell‐free bronchial wash (BW) and BAL fluid samples. Individual responses for each end‐point were compared using Wilcoxon‐signed rank tests. [Colour figure can be viewed at wileyonlinelibrary.com]

To confirm the validity of these initial *in vitro* findings to the *in vivo* context we explored whether similar CD8 priming occurred in human subjects after an experimental diesel challenge (300 μg/m^3^ particulate matter (PM10) of 10μm diameter or smaller PM_10_ for 1 hr, with bronchoscopy performed 6‐hr after exposure). Granzyme A mRNA was found to be elevated at this time‐point in BAL cell leucocytes following diesel challenge (Fig. 5a, *P* = 0·017), with immunohistochemistry demonstrating that protein expression was restricted to the lymphocytic population (Fig. 5b), with an increase in Granzyme A‐positive cells (Fig. 5c, *P* = 0·015). No corresponding increase in BW or BAL fluid Granzyme A was observed at 6 hr (Fig. 5d). Concentrations of Granzyme B and Perforin were also measured in the recovered BW and BAL fluid but were also not altered following the diesel challenge (data not shown).

## Discussion

In summary, our study shows that UPM stimulation of primary human CD1c^+^ mDCs enhances their maturation and activation, with increased expression of CD83 and the lymph‐node homing chemokine receptor CCR7, but not of MHC class I (HLA‐ABC). In allogeneic co‐cultures UPM treatment of CD1c^+^ mDCs significantly increased the proliferation of naive CD8 T lymphocytes, with significantly increased production of IFN‐*γ*, TNF‐*α*, IL‐13, and Granzymes A and B. Intracellular cytokine staining of expanded CD8 T lymphocytes showed significantly increased priming of IFN‐*γ*‐secreting CD8 T lymphocytes with UPM‐stimulated mDCs. Hence, overall, UPM treatment of mDCs enhanced their ability to prime pro‐inflammatory naive CD8 T lymphocytes in this *in vitro* culture system. Addition of GM‐CSF to UPM‐stimulated mDCs further enhanced naive T‐cell cytokine production and proliferation, despite diminishing MHC class I expression.

These findings are consistent with the known pro‐inflammatory effects of ambient air pollution *in vivo*,^30^ and expand our understanding of the effect of UPM on DC function and CD8 T‐lymphocyte responses. Induction of CCR7 on human mDCs by UPM, as we report, supports the results from murine experiments that diesel exhaust particle‐laden DCs translocate to lymph nodes after diesel exhaust particle instillation in to the lungs,^31^ and is important given that lymph nodes are the primary site for priming of new immune responses *in vivo*. Decreased expression of CCR2 and CCR6 on mDCs after exposure to UPM in the lungs as indicated by our results may act to release these mDCs from the pulmonary environment, as both are important in chemotaxis of inflammatory DCs in to the lungs.^32^, ^33^, ^34^ However, the resulting effects of down‐regulation of CCR2 and CCR6 on DCs are probably more complex – for example Sato and colleagues have previously shown CCR2 to have a role in migration of Langerhans cells (skin‐resident dendritic cells) to and within lymph nodes, and CCR2^−/−^ knockout mice to have an exaggerated T helper type 2 immune response to *Leishmania major* infection.^35^

Our novel finding of a pro‐inflammatory effect of UPM on DC‐stimulated CD8 T‐lymphocyte responses, important in the pathology of COPD, is broadly consistent with the literature studying the effect on CD4 T‐lymphocyte responses.^16^, ^17^, ^18^, ^19^ That such an effect is enhanced with GM‐CSF is in agreement with our current understanding for a pro‐inflammatory role for GM‐CSF in pollution‐induced lung disease.^16^, ^18^, ^19^ Although not the focus of this research, our finding of suppression of MHC class I expression by GM‐CSF is intriguing and deserves more detailed investigation in dedicated studies.

Respiratory tract infections underpin most exacerbations of airway diseases and exposure to air pollution is known to be associated with increased symptomatic respiratory infections.^5^, ^6^, ^7^ Possible mechanisms by which air pollution might impair anti‐microbial immune responses have been identified to explain this association.^36^, ^37^ Indeed our previous work with DC‐stimulated naïve CD4 T lymphocytes showed decreased priming of IFN*γ*‐producing CD4 T lymphocytes in similar experiments examining the effect of adding UPM to GM‐CSF‐treated mDCs.^16^ However, the present research with naive CD8 T lymphocytes, using a comparable experimental system, does not show UPM to impair priming of an IFN‐*γ*‐producing CD8 response, but in contrast demonstrates enhancement of this response.

Our earlier study demonstrated that IL‐6 contributed to the effect of UPM co‐activation of GM‐CSF‐treated mDCs on priming of naive CD4 T cells. One possible explanation of the differing effects of UPM on priming of IFN‐*γ* producers in naive CD4 and CD8 T‐lymphocyte mDC‐stimulated cultures could be differing sensitivity to IL‐6. Both CD4 and CD8 T lymphocytes express the IL‐6 receptor,^38^ and IL‐6 has been shown to be capable of enhancing antigen‐independent proliferation of CD8 T lymphocytes.^39^ However, in a limited number of experiments addition of an anti‐MHC class I antibody inhibited T‐cell cytokine expression in UPM‐stimulated cultures at odds with UPM stimulating naive CD8 T‐cell responses by an antigen‐independent mechanism (see Supplementary material, Fig. S2). CD8 and CD4 T lymphocytes are known to differ in their ease of activation and proliferation;^40^, ^41^ for example, the duration of antigen presentation necessary for lymphocyte response appears different for CD4 and CD8 T lymphocytes.^42^ Such differences may well underlie the contrasting effects of UPM–GM‐CSF‐treated mDCs on stimulation of naive CD4 and CD8 T‐lymphocyte responses.

Although cell culture studies are important in understanding mechanisms of disease, a limitation is that they cannot recapitulate the complex interaction between multiple different types of structural and immune cell as occurs after real‐life *in vivo* exposure to air pollution. Therefore, it is important that the capacity for UPM in this study to enhance lymphocyte responses is consistent with previous research including murine models and human studies. Murine studies by Saunders *et al*. in *Rag1* knockout and wild‐type mice have shown aspects of PM‐induced airways pathology to be lymphocyte dependent.^43^ Yoshizaki *et al*. have shown chronic diesel exhaust particle exposure in mice to lead to increased CD8 T cells within the lung together with alveolar airspace enlargement.^44^ Controlled human exposure to diesel exhaust and also exposure to traffic‐related air pollution have both shown increased numbers of lymphocytes within the lungs after pollution exposure.^29^, ^45^ Indeed we have found that controlled human exposure to diesel exhaust results in increase in cellular *GRZMA* mRNA expression in lower respiratory tract leucocytes, with immunocytochemistry indicating an increase in Granzyme A‐positive lymphocytes (Fig. 5). Increased secreted protein concentrations of Granzyme A, and other mediators studied, were not evident at 6 hr post‐exposure, but this probably reflects the very early time‐point assayed for a lymphocyte response.

Cytokine‐mediated and cytotoxic responses of CD8 Tc1 lymphocytes eradicate virus‐infected cells, but can also cause damage to bystander uninfected cells. Therefore adaptive immune responses are carefully regulated to prevent excessive cell damage.^12^ An action of air pollution PM to further increase pro‐inflammatory cytokine production by CD8 T lymphocytes responding to a viral infection may be detrimental by increasing bystander damage and pathological inflammation. Indeed, pathological CD8 T‐lymphocyte responses are increasingly thought to contribute to lung damage in COPD.^12^, ^46^ For example, Makris *et al*. found an increased frequency of CD8 T lymphocytes in induced sputum from COPD patients at the time of exacerbation, interestingly with a decreased frequency of CD4 T lymphocytes at exacerbation.^47^ Similarly CD8 T‐lymphocyte responses may contribute to pathology in asthma.^48^

Interestingly, McKendry *et al*. recently showed that upon *ex vivo* infection of resected lung tissue with influenza A virus, there was significantly greater IFN‐*γ* production by explants from patients with COPD than from healthy controls,^49^ suggesting that inadequate IFN‐*γ* production is not the cause of pathological inflammation in viral infections in patients with COPD.^49^

Granzymes can induce targeted cell death through diverse mechanisms, which can aid viral clearance. However, granzymes also exhibit non‐cytotoxic functions, with proposed roles in chronic inflammation, and impaired wound healing.^50^ For example, Granzyme A can stimulate pulmonary fibroblasts to secrete IL‐6 and CXCL8.^51^ Granzyme B secreting T cells in particular are thought to contribute to the pathogenesis of COPD^52^, ^53^ and our novel finding that UPM‐treatment enhanced Granzyme secretion supports UPM‐induced CD8 T‐cell responses being pro‐inflammatory to the airways. The relevance of UPM‐mediated up‐regulation of granzymes, and their potential cytotoxic and/or non‐cytotoxic roles in UPM‐mediated exacerbations of chronic airway diseases are important topics for future research, which could be explored initially through assessing a wider range of CD8 T‐cell functions including CD107a, a marker of cytotoxic degranulation, and Fas‐ligand‐induced apoptosis of target cells.

In summary, exposure to air pollution particulate matter enhances multiple facets of CD1c^+^ myeloid DC maturation, including up‐regulation of lymph‐node homing CCR7. Particulate matter also enhances the ability of mDCs to prime naive CD8 T lymphocytes with increased lymphocyte proliferation and production of pro‐inflammatory cytokines. An action of ambient PM to perturb adaptive CD8 T‐lymphocyte responses may cause pathological inflammation and contribute to the association between exposure to air pollution and exacerbations of airways diseases.

## Disclosures

The authors have no conflicting interests to declare.

## Supporting information


**Figure. S**1. Myeloid dendritic cell (mDC) expression of chemokine receptors as measured by quantitative real‐time PCR. Effect of 5 μg/ml urban particulate matter (UPM) with/without 50 ng/ml granulocyte–macrophage colony‐stimulating factor (GM‐CSF) on mDC expression of chemokine receptor genes in 20‐hr cultures, relative to the housekeeping gene *18S*, as measured by quantitative real‐time PCR. Two‐way analyses of variance, *n* = 5.

**Figure. S**2. Inhibition of urban particulate matter (UPM) ‐induced naive CD8 T‐cell response with anti‐MHC class I blockade. Flow‐cytometry contour plots (Forward scatter (FSc‐A) as a measure of lymphocyte blasting against intracellular interferon‐*γ* staining) for naive CD8 T cells stimulated by UPM‐treated myeloid dendritic cell (mDCs) in the presence of an anti‐MHC class I antibody or isotype control, after 5 days co‐culture and 2 days expansion.

**Appendix S1**. Supplementary methods for human exposure studies.
