# Supplementary material for: Urban particulate matter stimulation of human dendritic cells enhances priming of naive CD8 T lymphocytes
Source: Immunology. 2017 Nov 28;153(4):502–12. doi: 10.1111/imm.12852 (PMC5838419; doi:10.1111/imm.12852)
Supplement: Supplementary file 1 — Figure. S1. Myeloid dendritic cell (mDC) expression of chemokine receptors as measured by quantitative real‐time PCR. Effect of 5 μg/ml urban particulate matter (UPM) with/without 50 ng/ml granulocyte–macrophage colony‐stimulating factor (GM‐CSF) on mDC expression of chemokine receptor genes in 20‐hr cultures, relative to the housekeeping gene 18S, as measured by quantitative real‐time PCR. Two‐way analyses of variance, n = 5. [file IMM-153-502-s001.pdf]

A

Control GM-CSF

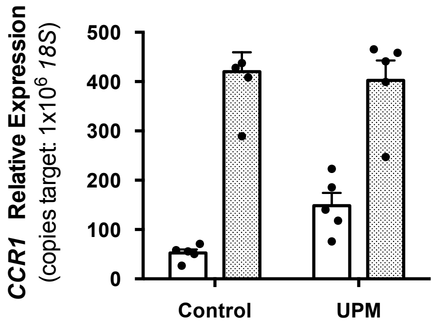

Source of Variation	P value
+/- UPM	0.1252
+/- GM-CSF	0.0005

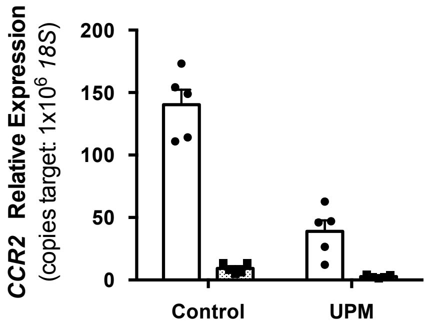

Source of Variation	P value
+/- UPM	0.0059
+/- GM-CSF	< 0.0001

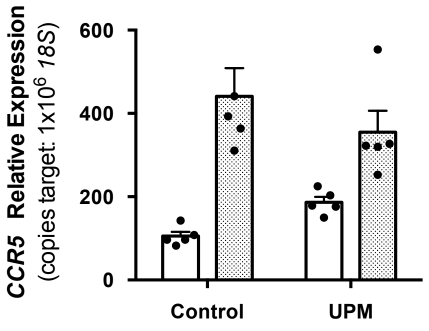

Source of Variation	P value
+/- UPM	0.8804
+/- GM-CSF	0.0091

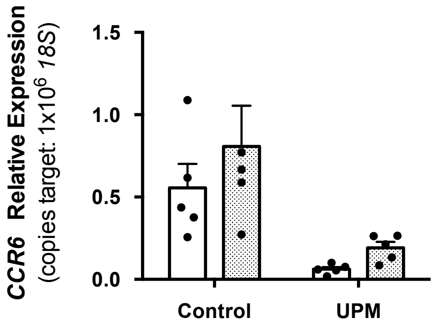

Source of Variation	P value
+/- UPM	0.0059
+/- GM-CSF	0.3142

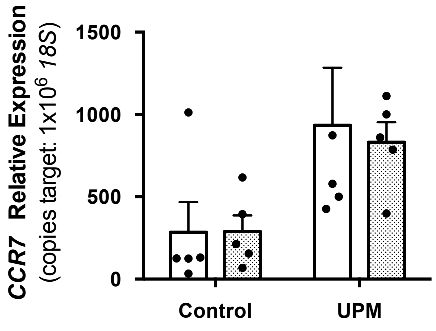

Source of Variation	P value
+/- UPM	0.0129
+/- GM-CSF	0.8184

Suppl Figure 1
