# Supplementary figures and images for: Urban particulate matter stimulation of human dendritic cells enhances priming of naive CD8 T lymphocytes
Source: Immunology. 2017 Nov 28;153(4):502–12. doi: 10.1111/imm.12852 (PMC5838419; doi:10.1111/imm.12852)

A

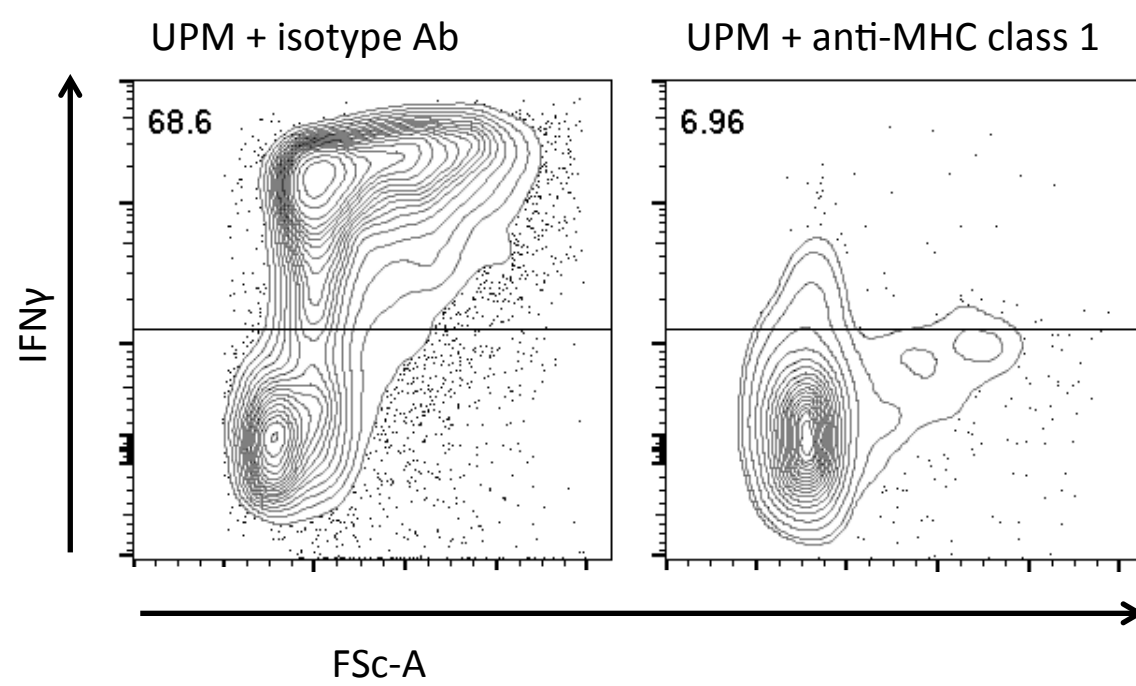

B

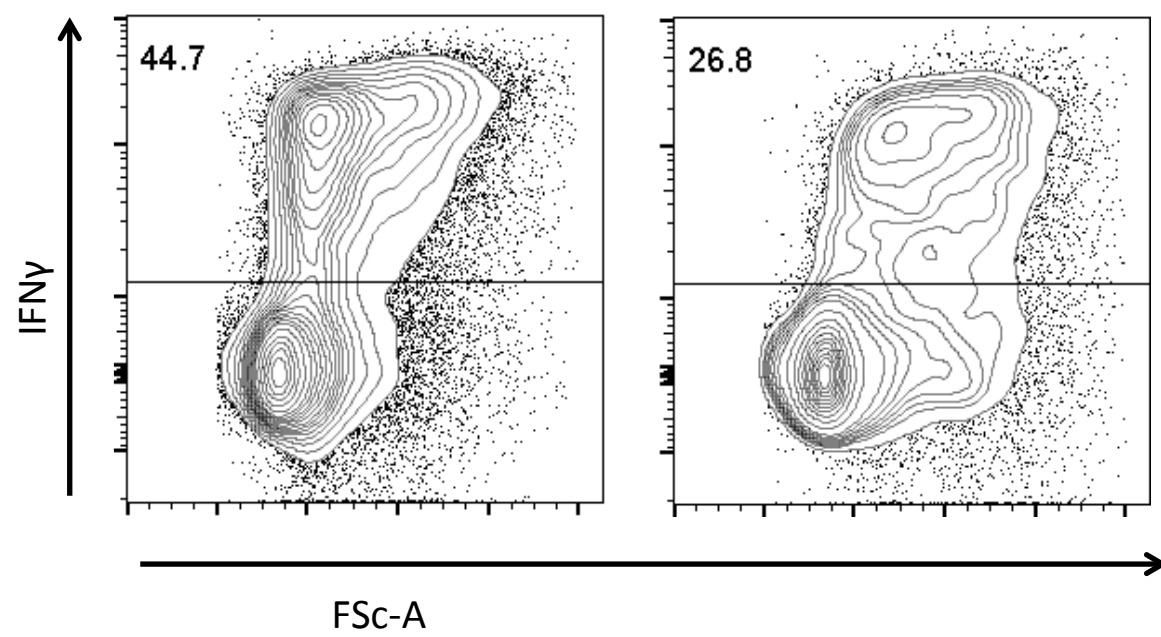

Supplement: Supplementary file 2 — Figure. S2. Inhibition of urban particulate matter (UPM) ‐induced naive CD8 T‐cell response with anti‐MHC class I blockade. Flow‐cytometry contour plots (Forward scatter (FSc‐A) as a measure of lymphocyte blasting against intracellular interferon‐γ staining) for naive CD8 T cells stimulated by UPM‐treated myeloid dendritic cell (mDCs) in the presence of an anti‐MHC class I antibody or isotype control, after 5 days co‐culture and 2 days expansion. [file IMM-153-502-s002.pdf]
