# Supplementary material for: Urban particulate matter stimulation of human dendritic cells enhances priming of naive CD8 T lymphocytes
Source: Immunology. 2017 Nov 28;153(4):502–12. doi: 10.1111/imm.12852 (PMC5838419; doi:10.1111/imm.12852)
Supplement: Supplementary file 3 — Appendix S1. Supplementary methods for human exposure studies. [file IMM-153-502-s003.docx]

**Urban particulate matter stimulation of human dendritic cells enhances priming of naïve CD8 T lymphocytes**

Running Title: PM enhances naïve CD8 responses

Paul E Pfeffer *et al.*

**Supplementary Methods for Human Exposure Studies**

**Diesel exhaust exposure and bronchoscopy**

Sixteen healthy non-smoking volunteers, free from respiratory infection or pre-existing allergic disease, were recruited into the diesel exposure study: 9 females and 7 males, mean age 24 years, range 20-31. Each subject was exposed on two separate occasions, once to filtered air and once to diesel engine exhaust, with exposures separated by at least three weeks to limit carry-over effects. Each exposure lasted for one hour, during which the subjects alternated between fifteen-minute of rest and exercise (20 L/min/m^2^ body surface). Diesel exhaust (DE) was generated by an idling Volvo diesel engine Volvo (TD45, 4.5 L, 4 Cylinders, 1991, 680 rpm) running on Gasoil E10 (Preem, Sweden) and diluted with filtered air to achieve a mean concentration PM_10_ in the chamber of 290 ± 27 µg/m^3^; associated of 2.9 ± 0.37 ppm NO, 0.84 ± 0.10 ppm NO_2_, 1.2 ± 0.15 ppm total hydrocarbons and 2.4 ± 0.53 ppm CO. This exposure duplicated an earlier protocol used to investigate the pro-inflammatory nature of diesel exhaust (1). The study was approved by the local Ethical Review Board at Umeå University, and performed in accordance with the Declaration of Helsinki with written informed consent of all participating volunteers.

Bronchoscopy was performed six-hours after the diesel and filtered air exposures using a flexible video bronchoscope (Olympus BF IT160, Tokyo, Japan) with proximal and lower airway samples obtained by bronchial wash (BW, 2 x 20 ml) and bronchoalveolar lavage (BAL, 3 x 60 ml) respectively, using sterile saline. All lavage samples were filtered through a nylon filter (pore diameter 100 µm) and centrifuged at 400 g for 15 minutes to obtain a cell free sample. Cell pellets were re-suspended in PBS at a cell concentration of 10^6^ cells/ml for total and differential cells counts (not reported here). A further 1ml aliquot was taken and centrifuged as above to pellet cells and the supernatant discarded. 1 ml RNALater was then added to the cell pellet which was stored at 4˚C for 24 hours before transfer to a -80˚C freezer for longer term storage.

**Mediator analysis**

Granzyme A was determined in cell free BW and BAL fluid samples using a commercial ELISA kit (BioVendor, Brno, Czech Republic), with a limit of sensitivity 0.4 pg/ml. Granzyme B and Perforin were measured using ELISA kits from Abcam (Cambridge, UK) with sensitivity (minimum detectable concentration) limits of 20 pg/ml and 40 pg/ml, respectively. All assays were performed according the manufacturers’ protocols.

**PCR analysis**

BAL leukocytes were lysed using homogenizers from Invitrogen Technologies and RNA isolated using the Qiagen RNeasy Mini kit, following manufacturer’s instructions in both cases. RNA concentration was determined in 1 μl of sample, using a Nanodrop ND-1000 spectrophotometer and the quality assessed with the RNA Nano 6000 Series II Agilent 2100 Bioanalyser assay, following manufacturer’s instructions. RNA was reverse transcribed into cDNA for qPCR using the Superscript III First-Strand Synthesis System for qPCR kit from Invitrogen Technologies (Paisley, UK), following manufacturer’s instructions. Glyceraldehyde 3-phosphate dehydrogenase (GAPDH) was selected as a reference gene for this data set using the geNORM Housekeeping Gene Selection Kit from PrimerDesign Ltd (Southampton, UK). qPCR analysis was carried out using gene-specific Taqman Gene Expression Assays from Applied BioSciences (Carlsbad, CA, US). cDNA was diluted 20-fold with PCR-grade water, and analysed in triplicate with no-template control, GAPDH as a reference gene and a 4-point standard curve to monitor the efficiency and reproducibility of the assay. Standards were created by pooling a small volume of undiluted cDNA from each sample in the data set and making three 4-fold dilutions with PCR-grade water.

**Immunocytochemistry**

BAL cell cytospins were fixed in 2% paraformaldehyde for 10 minutes prior to rinsing twice for 15 minutes in 15% sucrose prior to storage at -80 until required for staining. Tris-buffered saline (TBS) with 0,1 % Triton X-100 (TBST) was then applied for 30 minutes to permeabilizing cells. The endogenous peroxidases were inhibited using 0.1 % sodium azide and 0.3% hydrogen peroxide in distilled water. After washing 3 x 5 minutes in 0.1% TBST, nonspecific antibody binding was blocked with undiluted culture medium (DMEM, Sigma Aldrich) containing 10 % fetal calf serum and 1% bovine serum albumin for 30 minutes. The primary monoclonal antibody (Granzyme A, R&D systems, Abingdon, UK) was diluted in 0.05% TBST with 1% BSA applied and incubated overnight. The antibodies were then rinsed with 0.1% TBST 3 x 5 minutes, the biotinylated rabbit anti mouse (IgG F(ab)2; Dako, Glostrup, Denmark) applied and incubated for 2 hours. After further rinsing 3 x 5 minutes, streptavidin-biotin-horseradish peroxidase complex (Dako) was added and incubated for 2 hours. The Immunoreactivity of antibodies were visualized with diaminobenzidine to yield a brown color and counterstained with Mayer’s hematoxylin. Data were expressed as the % positive cells in each cytospin.

1. Salvi S, Blomberg A, Rudell B, Kelly F, Sandstrom T, Holgate ST, et al. Acute inflammatory responses in the airways and peripheral blood after short-term exposure to diesel exhaust in healthy human volunteers. Am J Respir Crit Care Med. 1999;159:702-9.
